# The association between the socioeconomic deprivation level and ischemic heart disease mortality in Japan: an analysis using municipality-specific data

**DOI:** 10.4178/epih.e2022059

**Published:** 2022-07-14

**Authors:** Tasuku Okui, Tetsuya Matoba, Naoki Nakashima

**Affiliations:** 1 Medical Information Center, Kyushu University Hospital, Fukuoka, Japan; 2 Department of Cardiovascular Medicine, Kyushu University, Fukuoka, Japan

**Keywords:** Coronary artery disease, Mortality, Japan

## Abstract

**OBJECTIVES:**

Geographical variation in the standardized mortality ratio (SMR) for ischemic heart disease (IHD) among municipalities has not been assessed in Japan. Additionally, associations between area-level socioeconomic deprivation indices and IHD mortality have not been identified in Japan. The present study investigated this association.

**METHODS:**

Information on IHD mortality was extracted from Vital Statistics data from 2018 to 2020 for each municipality in Japan. The socioeconomic deprivation level was derived from multiple socioeconomic characteristics. We classified municipalities into quintiles based on the deprivation level and investigated the association between the deprivation level and the SMR of IHD. Additionally, a Bayesian spatial regression model was used to investigate this association, adjusting for other municipal characteristics.

**RESULTS:**

Geographical variation in the SMR of IHD was revealed, and municipalities with high SMRs were spatially clustered. There was a weak negative correlation between the socioeconomic deprivation level and the SMRs (correlation coefficient, -0.057 for men and -0.091 for women). In contrast, the regression analysis showed a statistically significant positive association between deprived areas and the IHD mortality rate, and the relative risks for the most deprived municipalities compared with the least deprived municipalities were 1.184 (95% credible interval [CrI], 1.110 to 1.277) and 1.138 (95% CrI, 1.048 to 1.249) for men and women, respectively.

**CONCLUSIONS:**

A weak negative correlation between the socioeconomic deprivation level and the SMR was observed in the descriptive analysis, while the regression analysis showed that living in deprived areas was statistically positively associated with the IHD mortality rate.

## INTRODUCTION

Japan has among the highest life expectancies worldwide, and the life expectancy in Japan continues to increase [1]. Many factors are associated with the increased life expectancy; however, the main reason is the decline in mortality rates of major diseases in each age group over time [2]. In 2020, the leading cause of death in Japan was cancer, followed by heart disease [2], and the mortality rates of these diseases surpassed those of others. Ischemic heart disease (IHD) is the most common cause of death worldwide [3,4], and also accounts for a large proportion of heart disease-related deaths in Japan. The age-standardized mortality rate from IHD has decreased in Japan for years [5]; however, it remains a major cause of death in Japan, resulting in more than 60,000 deaths in 2020 [2]. The social costs of IHD, including morbidity and mortality-associated costs, increased between 1996 and 2014 because of population aging [6]. Therefore, preventive measures against IHD need to be considered in Japan.

Previous studies have shown that socioeconomic status was related to IHD incidence and mortality in other countries [7-10], and lower socioeconomic status tends to be associated with higher IHD incidence and mortality. Area-level socioeconomic deprivation has often been used as a socioeconomic status indicator. Some studies have shown an association between area-level socioeconomic deprivation and IHD incidence or mortality in other countries [11-13]. Area-level socioeconomic deprivation was also found to be positively associated with the incidence and mortality of stroke and cardiovascular diseases in Japan [14-16].

In contrast, nationwide studies have shown that a higher IHD mortality rate was observed in high-occupational classes, such as white-collar workers or administrative and managerial workers, in Japan [17,18]. Additionally, an ecological study showed that the standardized mortality ratio (SMR) of IHD was lower in municipalities with lower socioeconomic positions [19]. Therefore, some previous studies using nationwide data in Japan have shown that high, not low, socioeconomic status was associated with high IHD mortality. However, these studies using Vital Statistics records did not adjust for other characteristics in the analysis. Moreover, spatial autocorrelation of IHD mortality among municipalities was not considered in the ecological analysis [19]. It will be meaningful to reinvestigate this association by addressing those limitations of previous research. Moreover, geographic variation in IHD mortality among municipalities in Japan has not been revealed, and it is not yet known which, if any, municipalities have disproportionately high SMRs of IHD.

Therefore, this study identified geographic variation in IHD mortality and investigated the association between area-level socioeconomic deprivation and IHD mortality by a spatial statistics model using Vital Statistics records in Japan.

## MATERIALS AND METHODS

### Data

Vital Statistics data of Japan from 2018 to 2020 were used [2]. The Vital Statistics data are the official government statistics in Japan, and this resource tallies the number of births, deaths, marriages, and other demographic phenomena. The IHD mortality data by gender, year, and age group in all of Japan and the IHD mortality data by municipality were used to calculate the SMR for each municipality. The International Classification of Diseases-10 codes corresponding to IHD mortality are I20 to I25. The population data were obtained from “the survey of the population, demographics, and households based on the basic resident registry” [20].

Japan comprises 8 regions (Hokkaido, Tohoku, Kanto, Chubu, Kinki, Chugoku, Shikoku, and Kyushu), which are divided into 47 prefectures. Each prefecture contains municipalities comprising cities, wards, towns, and villages. Each of the government-designated cities and the 23 wards in Tokyo was treated as 1 municipality, and data from all 1,741 municipalities in Japan were included.

Area-level socioeconomic deprivation is usually derived from multiple areal socioeconomic characteristics [21-23]. An indicator derived by applying principal component analysis to municipal socioeconomic characteristics in Japan was used as the index of area-level socioeconomic deprivation in this study [22]. The deprivation level can be calculated by the following equation, where all variables are standardized.

Deprivation level= 0.517× proportion of fatherless households+ 0.110× proportion of low educational level+0.470× proportion of unemployed persons+0.591 × proportion of divorced persons+ 0.201× proportion of laborers−0.152× proportion of households living in owner-occupied housing−0.293 × taxable income per capita.

A high deprivation level of a municipality indicates a high proportion of individuals with a lower socioeconomic status. The data obtained from the census and survey on the municipal taxation status conducted by the Ministry of Internal Affairs and Communications were used to calculate the deprivation level [24,25].

Other municipal characteristics associated with regional differences in IHD or acute myocardial infarction (AMI) mortality in Japan were also used in the analysis [26,27]. Specifically, we used the number of emergency hospitals per capita, the number of medical clinics per capita, the number of physicians per capita, population density, the number of births per capita, and the proportion of workers engaged in the secondary sector of industries (mining, manufacturing, and construction). The proportion of workers engaged in the tertiary sector of industries was positively associated with IHD mortality [27]; however, this variable was not used herein because it was strongly correlated with the proportion of workers engaged in the secondary sector of industries. The numbers of designated emergency hospitals and medical clinics were obtained from a survey on medical institutions, and the number of physicians was obtained from statistics on physicians, dentists, and pharmacists [24,28]. Data on the total area for each municipality were obtained from the municipal area statistics of Japan published by the geospatial information authority of the Ministry of Land, Infrastructure, Transport, and Tourism [24]. The number of births was obtained from the Vital Statistics, and the number of workers engaged in the secondary industries was obtained from the Census [24]. Moreover, we used the proportion of young people (persons aged less than 30 years old) in the analysis.

During this study, data in 2020 from the Census; the survey on medical institutions; the municipal area statistics of Japan; the statistics of physicians, dentists, and pharmacists; data from the survey on the municipal taxation status; and the Vital Statistics data were publicly available and used in the analysis. However, data on the proportion of laborers in 2020 were unavailable; hence, values from 2015 was used for that variable. Additionally, map data of Japan by municipalities were obtained from the digital national land information published by the Ministry of Land, Infrastructure, Transport, and Tourism [29].

### Statistical analysis

An ecological study was conducted to investigate the association between socioeconomic deprivation level and IHD mortality in each municipality. The mortality rate of IHD for each age group and gender in Japan was calculated using data from 2018 to 2020. We calculated the expected number of IHD deaths for each municipality and gender by multiplying the mortality rate by the population count for each age group, gender, and municipality. Additionally, an adjacency matrix for municipalities was derived for the spatial models.

The SMR was derived using empirical Bayesian methods with IHD mortality and the expected number of IHD deaths [30]. The SMRs were mapped to show geographical variation in IHD mortality in Japan. Moreover, municipalities were classified into quintiles based on the socioeconomic deprivation level, and the IHD mortality rate and the SMR were summarized depending on the quintiles.

A non-spatial Poisson regression model was used to investigate the association. IHD mortality was anticipated to follow the Poisson distribution, and the expected number of IHD mortality was used as an offset term in the non-spatial Poisson regression model. The socioeconomic deprivation level and other predictors were used as explanatory variables, and all the variables were standardized before the regression analysis. The relative risk (RR), 95% confidence interval, and p-values were calculated. Moran’s I statistics were calculated for the regression model residuals to test the spatial autocorrelation among residuals. Next, a Bayesian spatial regression model, called the Besag-York-Mollié model, was used to take into account the spatial autocorrelation of IHD mortality [31]. The posterior mean of the RR and its 95% credible intervals (CrI) were calculated for each variable.

Only municipalities that were adjacent to other municipalities were used in the association analysis. Moreover, several municipalities with extremely small populations because of an evacuation due to the Great East Japan Earthquake were not included in the association analysis. All the analyses were conducted using R version 4.1.3 (https://cran.r-project.org/).

### Ethics statement

Institutional review board approval was not required for this study because only publicly available data were analyzed in this study. All methods were carried out in accordance with relevant guidelines and regulations.

## RESULTS

**Figure 1.**
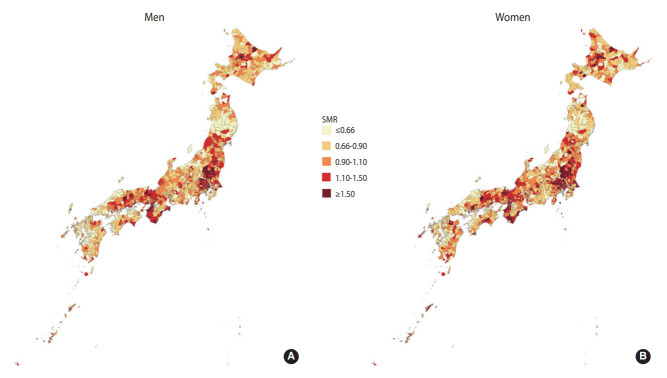
Geographical differences in the standardized mortality ratio (SMR) of ischemic heart disease in Japan (A: men, B: women).

Figure 1 shows the geographical variation in the SMR of IHD in Japan. Geographical variation was relatively similar between the genders. The SMRs tended to be high in the Kanto and Kinki regions, and municipalities with high SMRs were clustered.

Supplementary Material 1 shows the locations of the 8 regions in Japan.

Table 1 shows the municipalities with the highest SMRs of IHD. There were some overlapping areas with high SMRs in both genders.

Table 2 shows a summary of the municipal characteristics. There are 1,741 municipalities in Japan, of which 1,687 municipalities were included in the association analysis.

Supplementary Material 2 shows the municipalities with the highest levels of deprivation. Several municipalities in Fukuoka prefecture appeared as deprived municipalities.

**Figure 2.**
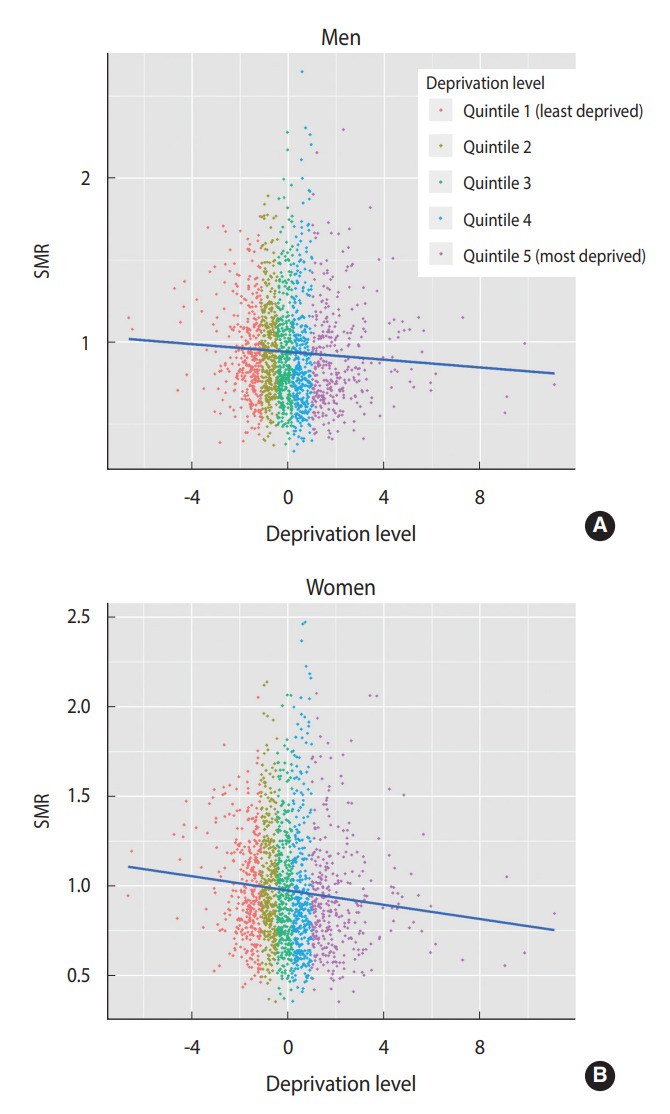
Scatterplot between the standardized mortality ratio (SMR) of and the deprivation level (A: men, B: women). The lines in the figure are regression lines.

Figure 2 shows a scatterplot between the SMR of IHD and the deprivation level. The correlation coefficient between the SMR of IHD and the deprivation level was -0.057 and -0.091 for men and women, respectively. Therefore, there was a weak negative correlation between the SMR and the deprivation level.

Table 3 shows the summary statistics of the mortality rate and SMR of IHD depending on the area-level socioeconomic deprivation. The relationship between the median of SMRs and the deprivation quintiles was J-shaped. The median SMRs for the least deprived quintile were higher than those for the most deprived quintile.

Additionally, the skewness for the SMR was 1.050 for men and 0.992 for women, indicating right-skewness, which is related to the result that the median values for all quintiles were less than 0 (Table 3). In other words, the SMRs for more than half of municipalities were less than 0.

The descriptive analyses in Table 3 and Figure 2 do not consider other municipal factors in the evaluation of the association between IHD mortality and the socioeconomic deprivation level. Therefore, it is necessary to refer to the results of multivariate regression analysis.

Supplementary Material 3 shows the results of the non-spatial Poisson regression model. The socioeconomic deprivation level was positively associated with IHD mortality when adjusting for other characteristics. However, Moran’s I statistics for the Poisson regression residuals were 0.455 (p< 0.001) and 0.407 (p< 0.001) for men and women, respectively. Therefore, a spatial autocorrelation was found in the residuals, suggesting that it would be appropriate to conduct a further analysis using a spatial regression model.

Table 4 shows the results of the Bayesian spatial regression model. The socioeconomic deprivation level and IHD mortality were positively associated when adjusting for spatial autocorrelation and other municipal predictors, and a statistically significant association was observed in men. Additionally, the absolute value of the regression coefficient, which indicates the degree of association with the outcome, for the socioeconomic deprivation level was the largest among the explanatory variables.

Table 5 depicts the results of the Bayesian spatial regression model using quintiles of the socioeconomic deprivation level. The RR of quintile 5 (the most deprived) compared to quintile 1 (the least deprived) was 1.184 (95% CrI, 1.110 to 1.277) for men and 1.138 (95% CrI, 1.048 to 1.249) for women, indicating that the risk of IHD mortality for the most deprived areas was approximately 18% and 14% higher, respectively, than that of the least deprived areas when adjusting for other factors. Statistically, RRs in quintiles 2 and 4 were also significantly higher than those in quintile 1 in both genders.

Supplementary Material 4 shows the results of the Bayesian spatial regression model using each municipal socioeconomic characteristic that was utilized to derive the socioeconomic deprivation level. The proportion of divorced persons was statistically significantly and positively associated with IHD mortality in both genders.

## DISCUSSION

This study investigated the association between municipal socioeconomic deprivation levels and IHD mortality in Japan. The spatial regression analysis, considering other municipal characteristics, including population density, showed a statistically significantly higher RR of IHD mortality in the most deprived areas than in the least deprived areas for both genders.

In contrast, the median SMRs for the least deprived quintile were higher than those for the most deprived quintile. The result is consistent with a previous study in Japan, in which the municipal socioeconomic position was defined by the educational level and taxable income [19]. Population density is a possible reason for the high median SMRs in the least deprived quintile. The municipal socioeconomic deprivation level and population density were negatively correlated, and IHD mortality was high in urban areas of Japan [32].

The positive association between IHD mortality and socioeconomic deprivation level in the regression analysis might have resulted from relationships between low socioeconomic status and IHD risk factors. Lifestyle-related diseases or behaviors, such as hypertension, diabetes, dyslipidemia, obesity, smoking, and alcohol use, are recognized as IHD risk factors [33]. In particular, hypertension is among the largest risk factors for IHD [34]. An occupational cohort study in Japan [35] revealed that people with higher education or income levels had a lower risk of hypertension. A higher smoking rate, another major IHD risk factor [36] was positively associated with lower household income and educational levels in Japan [37,38]. Moreover, hypercholesterolemia, an IHD risk factor [39,40] was negatively associated with household expenditures among Japanese men [41]. Furthermore, a study in Japan showed that lower education and lower household expenditures were positively associated with ignorance of cardiovascular risk factors [42]. Additionally, lower socioeconomic status was associated with delays in prehospital and hospital treatment, which potentially affects mortality in patients with acute coronary syndrome, including AMI [43].

Furthermore, deprived neighborhoods may have an adverse effect on IHD mortality. An association between neighborhood deprivation and the incidence or management of IHD was previously shown in Sweden and Korea, and it has been discussed that living in deprived neighborhoods is related to a less health-promoting environment, as exemplified by factors such as safe places to exercise, stores selling healthy foods, or the availability of services provided to support daily lives [12,44]. It has also been pointed out that supportive neighborhoods are associated with higher physical activity and that higher perceptions of neighborhood safety or lower crime are associated with more walking [45]. Moreover, neighborhoods can affect cardiovascular risk through psychological stress or sleep quality [45]. Therefore, it is possible that deprived neighborhoods also affect people’s behaviors and health status in Japan. As another possible reason, a nationwide study by the Japanese Circulation Society (JCS) showed that the 30-day mortality of AMI complicated with cardiogenic shock was 42.3% in Japan from 2012 to 2016 [46]. The AMI mortality rate was related to institutional characteristics, such as the number of JCS-certified cardiologists or the use of a mechanical circulatory support system. These institutional characteristics might vary depending on the municipal deprivation level. Those hypotheses regarding possible reasons for the association between deprivation and IHD mortality need to be investigated in further studies.

As an implication of this study, the regression analysis showed that living in deprived areas was positively associated with IHD mortality rate. In contrast, some previous studies showed an association between higher socioeconomic status and higher IHD mortality [17-19]. However, as previous studies used the government statistics without adjusting for other factors, the results do not necessarily mean that higher socioeconomic status adversely affects IHD mortality. Taking into account the results of this study and the high prevalence of some risk factors among groups with lower socioeconomic status [35,37], it is considered that lower socioeconomic status adversely affects IHD. A further study investigating the association between IHD incidence or survival, and socioeconomic status is needed to understand the mechanisms of these associations.

This study has some limitations. First, because of the ecological study design, it is not certain that the deprived neighborhoods themselves had adverse effects on IHD mortality because the proportion of people with low socioeconomic status may have particularly affected regional differences in IHD mortality. Only aggregated data on IHD mortality were publicly available, and an analysis of the association taking into account both individual-level socioeconomic status and the municipal deprivation level was not conducted. Moreover, this was a cross-sectional study, and the causal relationship between the deprivation level and IHD mortality could not be assessed. For this purpose, further research using longitudinal data should be conducted. Third, the accuracy of the Vital Statistics records in reporting the causes of death in Japan is uncertain. A previous study indicated high sensitivity for IHD as a cause of death in a city in Japan, although its positive predictive value was not high [47]. Therefore, further validation should be conducted to assess the accuracy of IHD mortality as a cause of death in death certificates.

## Figures and Tables

**Table 1.** Municipalities with the highest standardized mortality ratio (SMR) of ischemic heart disease

Rank	Men		Women	
	Municipality name (prefecture name)	SMR	Municipality name (prefecture name)	SMR
1	Izumiotsu city (Osaka)	2.649	Choshi city (Chiba)	2.472
2	Choshi city (Chiba)	2.307	Shijonawate city (Osaka)	2.462
3	Kushimoto town (Wakayama)	2.295	Izumiotsu city (Osaka)	2.368
4	Kashiwara city (Osaka)	2.279	Ogose town (Saitama)	2.225
5	Kishiwada city (Osaka)	2.265	Kishiwada city (Osaka)	2.184
6	Daitou city (Osaka)	2.204	Daitou city (Osaka)	2.160
7	Izumi city (Osaka)	2.173	Motegi town (Tochigi)	2.138
8	Kaizuka city (Osaka)	2.156	Hachioji city (Tokyo)	2.121
9	Nasukarasuyama city (Tochigi)	2.112	Kaizuka city (Osaka)	2.075
10	Habikino city (Osaka)	1.999	Izumi city (Osaka)	2.065

**Table 2.** Summary statistics for municipal characteristics used in this study

Characteristics		Median (interquartile range) (n=1,687)
Characteristics used to derive the deprivation level		
	Taxable income per capita (1,000 yen)	1,219.1 (1,048.9-1427.4)
	Proportion of households living in owner-occupied housing	74.1 (65.6-82.3)
	Proportion of divorced people	5.3 (4.7-6.1)
	Proportion of fatherless households	1.2 (0.9-1.5)
	Proportion of people with low educational level	16.9 (12.0-23.6)
	Proportion of laborers	7.3 (6.5-8.4)
	Proportion of unemployed people	3.6 (3.0-4.2)
Other characteristics		
	Population density (population per hectare)	2.1 (0.6-8.2)
	Proportion of young people	24.2 (21.1-27.3)
	No. of births^1^	541.9 (419.3-664.4)
	Proportion of workers engaged in the secondary sector of industries	23.8 (18.3-29.2)
	No. of designated emergency hospitals^1^	2.5 (0.0-5.1)
	No. of medical clinics^1^	69.7 (55.1-87.0)
	No. of physicians^1^	136.7 (77.8-209.3)

1 Number per 100,000 population.

**Table 3.** Summary statistics of the mortality rate and SMR of ischemic heart disease depending on the areal socioeconomic deprivation level

Quintiles	Mortality rate^1^		SMR	
	Mean	Median (IQR)	Mean	Median (IQR)
Men				
Quintile 1 (least deprived)	68.4	64.6 (44.2-85.2)	0.949	0.911 (0.760-1.106)
Quintile 2	76.6	73.8 (53.1-93.6)	0.977	0.942 (0.760-1.136)
Quintile 3	72.2	64.3 (46.6-91.6)	0.929	0.834 (0.708-1.082)
Quintile 4	74.0	62.6 (43.0-95.5)	0.936	0.840 (0.676-1.094)
Quintile 5 (most deprived)	74.0	62.0 (46.6-89.8)	0.910	0.847 (0.695-1.064)
Women				
Quintile 1 (least deprived)	50.7	44.7 (28.8-61.3)	0.993	0.945 (0.768-1.195)
Quintile 2	54.7	49.0 (35.6-66.3)	1.016	0.959 (0.803-1.209)
Quintile 3	54.2	45.7 (32.7-67.9)	0.965	0.924 (0.730-1.141)
Quintile 4	53.2	46.5 (31.9-68.4)	0.971	0.868 (0.692-1.149)
Quintile 5 (most deprived)	51.8	45.1 (32.6-65.3)	0.927	0.886 (0.710-1.057)

SMR, standardized mortality ratio; IQR, interquartile range.

1 Mortality per 100,000 person-years.

**Table 4.** The results of the Bayesian spatial regression model showing the association between ischemic heart disease mortality and municipal characteristics

Explanatory variables^1^	Men	Women
Socioeconomic deprivation level	1.063 (1.031, 1.093)	1.029 (0.997, 1.063)
Population density	1.036 (1.009, 1.073)	1.017 (0.985, 1.061)
Proportion of young people	0.968 (0.919, 1.014)	0.999 (0.936, 1.054)
No. of births^2^	1.020 (0.982, 1.069)	1.002 (0.959, 1.059)
Proportion of workers engaged in the secondary sector of industries	0.995 (0.963, 1.026)	0.994 (0.962, 1.029)
No. of designated emergency hospitals^2^	1.020 (0.997, 1.044)	1.014 (0.985, 1.042)
No. of medical clinics^2^	0.995 (0.968, 1.020)	0.985 (0.954, 1.018)
No. of physicians^2^	0.996 (0.977, 1.015)	0.993 (0.972, 1.013)

Values are presented as relative risk (95% credible interval).

1 Standardized values were used for all explanatory variables.

2 Number per 100,000 population.

**Table 5.** The results of the Bayesian spatial regression model using quintiles of the socioeconomic deprivation level showing an association with ischemic heart disease mortality

Explanatory variables^1^	Men	Women
Socioeconomic deprivation level		
Quintile 1 (least deprived)	1.000 (reference)	1.000 (reference)
Quintile 2	1.095 (1.046, 1.160)	1.085 (1.019, 1.161)
Quintile 3	1.058 (1.001, 1.127)	1.040 (0.977, 1.124)
Quintile 4	1.108 (1.049, 1.192)	1.083 (1.010, 1.172)
Quintile 5 (most deprived)	1.184 (1.110, 1.277)	1.138 (1.048, 1.249)
Population density	1.041 (1.001, 1.081)	1.015 (0.977, 1.050)
Proportion of young people	0.975 (0.932, 1.019)	1.001 (0.956, 1.055)
No. of births^2^	1.012 (0.974, 1.052)	1.001 (0.956, 1.039)
Proportion of workers engaged in the secondary sector of industries	0.997 (0.969, 1.030)	0.990 (0.961, 1.022)
No. of designated emergency hospitals^2^	1.020 (0.995, 1.044)	1.012 (0.985, 1.041)
No. of medical clinics^2^	0.993 (0.966, 1.018)	0.983 (0.956, 1.012)
No. of physicians^2^	0.994 (0.976, 1.012)	0.991 (0.970, 1.011)

Values are presented as relative risk (95% credible interval).

1 Standardized values were used for all explanatory variables except for the socioeconomic derivation level.

2 Number per 100,000 population.

## SUPPLEMENTARY MATERIALS

Supplementary Material 1. A map indicating 8 regions in Japan.

Supplementary Material 2. Municipalities with the highest-level deprivation

Supplementary Material 3. The results of non–spatial Poisson regression model showing the association between ischemic heart disease mortality and municipal characteristics

Supplementary Material 4. The results of Bayesian spatial regression model using each of municipal socioeconomic characteristics showing an association with ischemic heart disease mortality.
