# Supplementary figures and images for: The association between the socioeconomic deprivation level and ischemic heart disease mortality in Japan: an analysis using municipality-specific data
Source: Epidemiol Health. 2022 Jul 14;44:e2022059. doi: 10.4178/epih.e2022059 (PMC9754915; doi:10.4178/epih.e2022059)

Supplementary Materials


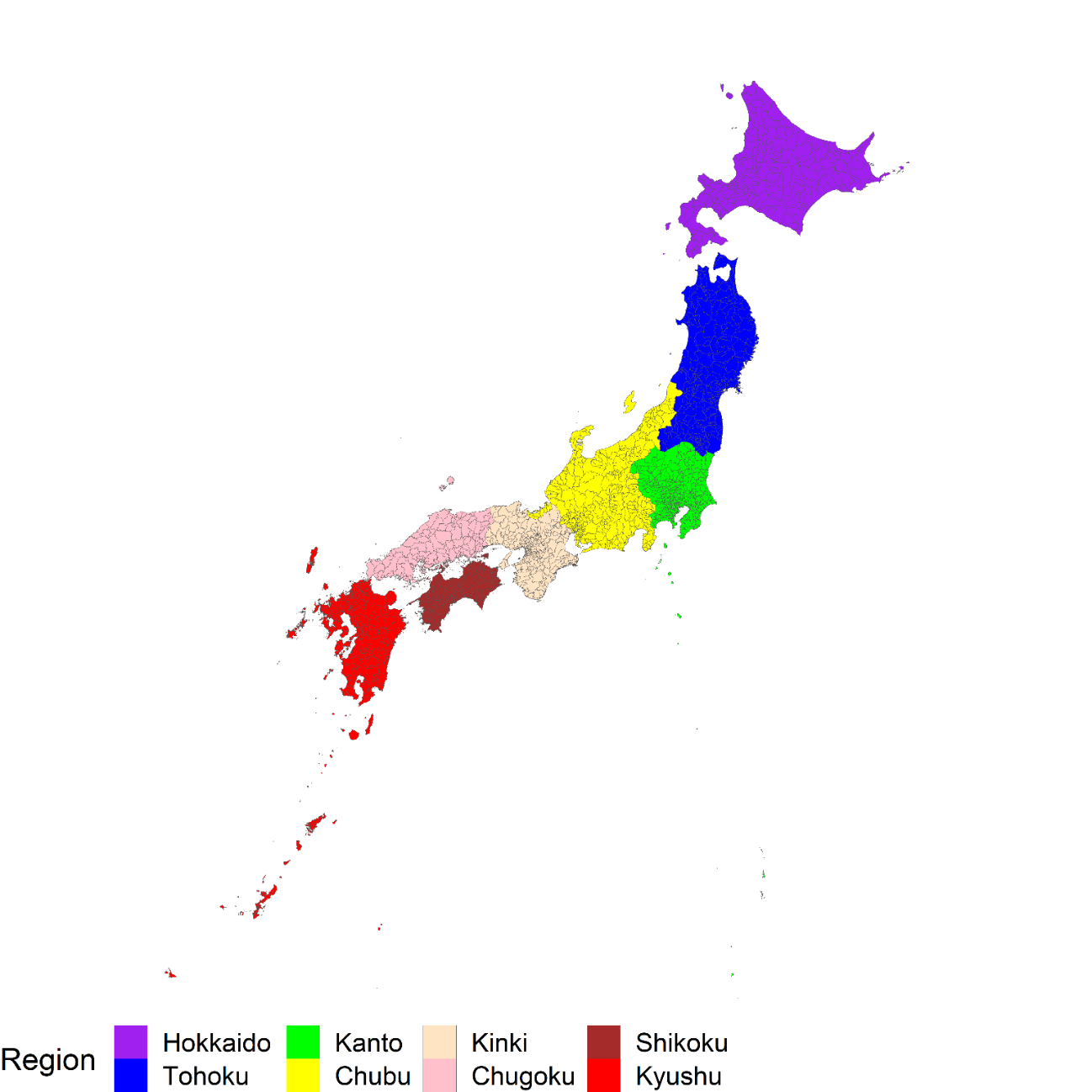
Supplementary Material 1. A map indicating 8 regions in Japan.

Supplement: Supplementary Material 1. — A map indicating 8 regions in Japan. [file epih-44-e2022059-suppl1.docx]
