# Supplementary material for: The association between the socioeconomic deprivation level and ischemic heart disease mortality in Japan: an analysis using municipality-specific data
Source: Epidemiol Health. 2022 Jul 14;44:e2022059. doi: 10.4178/epih.e2022059 (PMC9754915; doi:10.4178/epih.e2022059)
Supplement: Supplementary Material 2. — Municipalities with the highest-level deprivation [file epih-44-e2022059-suppl2.docx]

Supplementary Materials

Supplementary Material 2. Municipalities with the highest-level deprivation

| Rank | Municipality name (prefecture name) | Score of deprivation level |
| --- | --- | --- |
| 1 | Kawasaki town (Fukuoka) | 11.093 |
| 2 | Oto town (Fukuoka) | 9.855 |
| 3 | Fukuchi town (Fukuoka) | 9.110 |
| 4 | Itoda town (Fukuoka) | 9.031 |
| 5 | Kamisunagawa town (Hokkaido) | 7.275 |
| 6 | Kunigami village (Okinawa) | 6.149 |
| 7 | Kawara town (Fukuoka) | 5.953 |
| 8 | Tagawa city (Fukuoka) | 5.936 |
| 9 | Kin town (Okinawa) | 5.642 |
| 10 | Mizumaki town (Fukuoka) | 5.575 |
