# Supplementary material for: The association between the socioeconomic deprivation level and ischemic heart disease mortality in Japan: an analysis using municipality-specific data
Source: Epidemiol Health. 2022 Jul 14;44:e2022059. doi: 10.4178/epih.e2022059 (PMC9754915; doi:10.4178/epih.e2022059)
Supplement: Supplementary Material 3. — The results of non–spatial Poisson regression model showing the association between ischemic heart disease mortality and municipal characteristics [file epih-44-e2022059-suppl3.docx]

Supplementary Materials

Supplementary Material 3. The results of non–spatial Poisson regression model showing the association between ischemic heart disease mortality and municipal characteristics.

|  | Men | | Women | |
| --- | --- | --- | --- | --- |
| Explanatory variables | Relative risk (95% CI) | p-value | Relative risk (95% CI) | p-value |
| Socioeconomic deprivation level | 1.045 (1.037－1.054) | <0.001 | 1.015 (1.006－1.024) | 0.001 |
| Population density | 1.110 (1.106－1.115) | <0.001 | 1.090 (1.084－1.095) | <0.001 |
| Proportion of young people | 1.145 (1.125－1.165) | <0.001 | 1.142 (1.118－1.166) | <0.001 |
| No. of births* | 0.862 (0.849－0.875) | <0.001 | 0.877 (0.860－0.893) | <0.001 |
| Proportion of workers engaged in the secondary sector of industries | 1.036 (1.028－1.044) | <0.001 | 1.036 (1.027－1.046) | <0.001 |
| No. of designated emergency hospitals* | 1.069 (1.054－1.085) | <0.001 | 1.063 (1.046－1.081) | <0.001 |
| No. of medical clinics* | 1.016 (1.005－1.027) | 0.006 | 0.985 (0.971－0.998) | 0.025 |
| No. of physicians* | 0.990 (0.982－0.997) | 0.008 | 0.989 (0.980－0.998) | 0.016 |
| CI, confidence interval. Standardized values were used for all the explanatory variables. | | | | |
| * Number per 100,000 persons |  |  |  |  |
