# Supplementary material for: The association between the socioeconomic deprivation level and ischemic heart disease mortality in Japan: an analysis using municipality-specific data
Source: Epidemiol Health. 2022 Jul 14;44:e2022059. doi: 10.4178/epih.e2022059 (PMC9754915; doi:10.4178/epih.e2022059)
Supplement: Supplementary Material 4. — The results of Bayesian spatial regression model using each of municipal socioeconomic characteristics showing an association with ischemic heart disease mortality. [file epih-44-e2022059-suppl4.docx]

Supplementary Materials


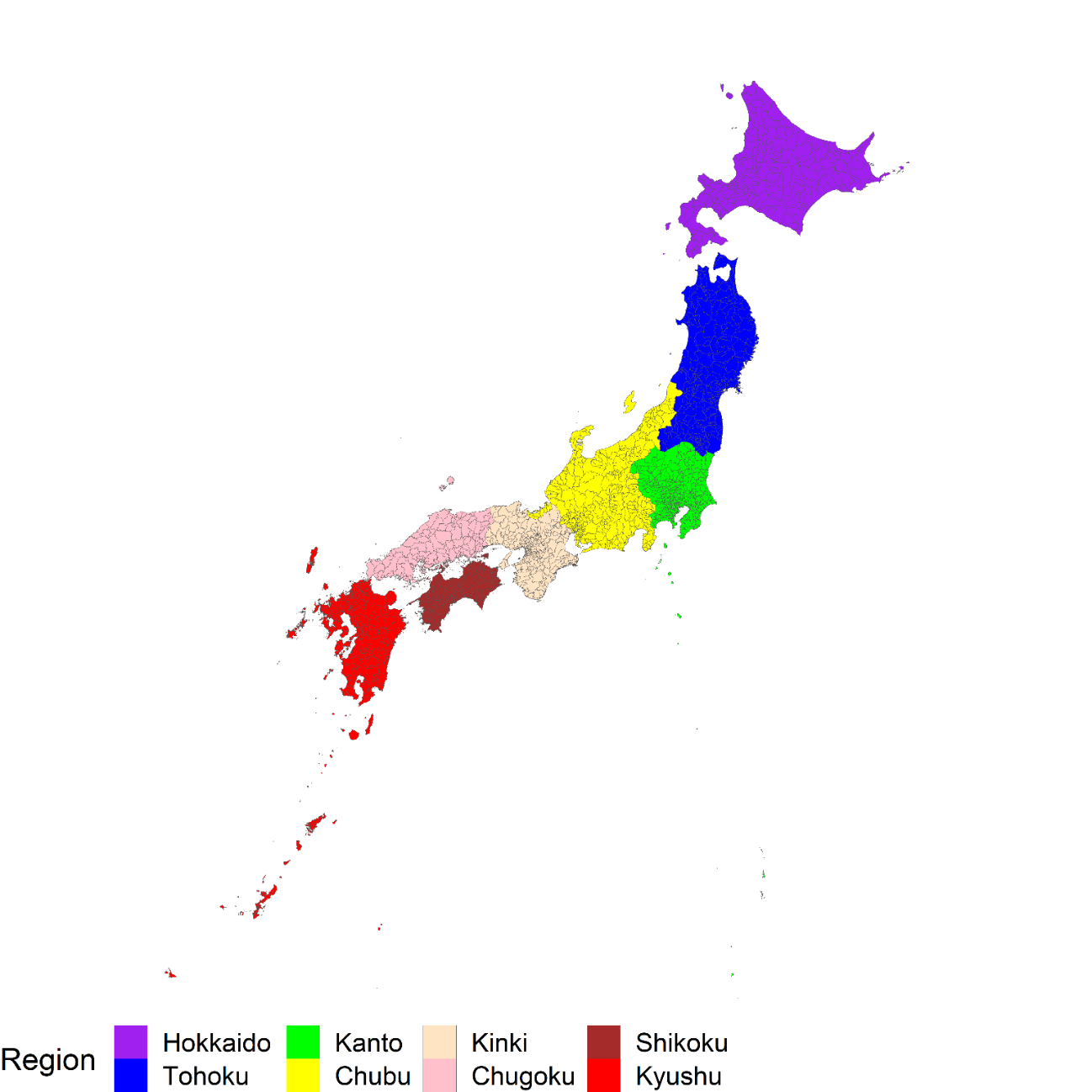
Supplementary Material 1. A map indicating 8 regions in Japan.

Supplementary Material 2. Municipalities with the highest-level deprivation

| Rank | Municipality name (prefecture name) | Score of deprivation level |
| --- | --- | --- |
| 1 | Kawasaki town (Fukuoka) | 11.093 |
| 2 | Oto town (Fukuoka) | 9.855 |
| 3 | Fukuchi town (Fukuoka) | 9.110 |
| 4 | Itoda town (Fukuoka) | 9.031 |
| 5 | Kamisunagawa town (Hokkaido) | 7.275 |
| 6 | Kunigami village (Okinawa) | 6.149 |
| 7 | Kawara town (Fukuoka) | 5.953 |
| 8 | Tagawa city (Fukuoka) | 5.936 |
| 9 | Kin town (Okinawa) | 5.642 |
| 10 | Mizumaki town (Fukuoka) | 5.575 |

Supplementary Material 3. The results of non–spatial Poisson regression model showing the association between ischemic heart disease mortality and municipal characteristics.

|  | Men | | Women | |
| --- | --- | --- | --- | --- |
| Explanatory variables | Relative risk (95% CI) | p-value | Relative risk (95% CI) | p-value |
| Socioeconomic deprivation level | 1.045 (1.037－1.054) | <0.001 | 1.015 (1.006－1.024) | 0.001 |
| Population density | 1.110 (1.106－1.115) | <0.001 | 1.090 (1.084－1.095) | <0.001 |
| Proportion of young people | 1.145 (1.125－1.165) | <0.001 | 1.142 (1.118－1.166) | <0.001 |
| No. of births* | 0.862 (0.849－0.875) | <0.001 | 0.877 (0.860－0.893) | <0.001 |
| Proportion of workers engaged in the secondary sector of industries | 1.036 (1.028－1.044) | <0.001 | 1.036 (1.027－1.046) | <0.001 |
| No. of designated emergency hospitals* | 1.069 (1.054－1.085) | <0.001 | 1.063 (1.046－1.081) | <0.001 |
| No. of medical clinics* | 1.016 (1.005－1.027) | 0.006 | 0.985 (0.971－0.998) | 0.025 |
| No. of physicians* | 0.990 (0.982－0.997) | 0.008 | 0.989 (0.980－0.998) | 0.016 |
| CI, confidence interval. Standardized values were used for all the explanatory variables. | | | | |
| * Number per 100,000 persons |  |  |  |  |

Supplementary Material 4. The results of Bayesian spatial regression model using each of municipal socioeconomic characteristics showing an association with ischemic heart disease mortality.

|  | Men | Women |
| --- | --- | --- |
| Explanatory variables | Relative risk (95% CrI) | Relative risk (95% CrI) |
| Analysis using taxable income per capita |  |  |
| Taxable income per capita | 0.962 (0.932－0.996) | 0.976 (0.941－1.009) |
| Population density | 1.046 (1.011－1.082) | 1.024 (0.988－1.057) |
| Proportion of young people | 0.989 (0.949－1.036) | 1.002 (0.958－1.060) |
| No. of births* | 1.017 (0.979－1.056) | 1.008 (0.966－1.047) |
| Proportion of workers engaged in the secondary sector of industries | 1.002 (0.973－1.033) | 0.999 (0.965－1.031) |
| No. of designated emergency hospitals* | 1.024 (0.999－1.049) | 1.014 (0.987－1.041) |
| No. of medical clinics* | 1.008 (0.981－1.037) | 0.993 (0.963－1.020) |
| No. of physicians* | 1.000 (0.981－1.019) | 0.996 (0.976－1.017) |
| Analysis using proportion of persons with low educational level |  |  |
| Proportion of persons with low educational level | 1.010 (0.965－1.052) | 1.029 (0.987－1.074) |
| Population density | 1.046 (1.018－1.082) | 1.024 (0.986－1.057) |
| Proportion of young people | 0.989 (0.933－1.030) | 1.019 (0.964－1.072) |
| No. of births* | 1.006 (0.975－1.047) | 0.998 (0.959－1.039) |
| Proportion of workers engaged in the secondary sector of industries | 1.001 (0.970－1.028) | 0.991 (0.956－1.027) |
| No. of designated emergency hospitals* | 1.023 (0.996－1.049) | 1.012 (0.986－1.040) |
| No. of medical clinics* | 0.994 (0.968－1.021) | 0.983 (0.953－1.011) |
| No. of physicians* | 0.996 (0.979－1.015) | 0.994 (0.974－1.015) |
| Analysis using proportion of fatherless households |  |  |
| Proportion of fatherless households | 1.034 (1.000－1.064) | 1.014 (0.985－1.048) |
| Population density | 1.045 (1.005－1.084) | 1.019 (0.980－1.055) |
| Proportion of young people | 0.966 (0.926－1.015) | 0.993 (0.935－1.045) |
| No. of births* | 1.009 (0.970－1.046) | 1.001 (0.959－1.048) |
| Proportion of workers engaged in the secondary sector of industries | 0.999 (0.967－1.030) | 0.994 (0.965－1.026) |
| No. of designated emergency hospitals* | 1.023 (0.997－1.046) | 1.014 (0.986－1.040) |
| No. of medical clinics* | 0.993 (0.969－1.019) | 0.982 (0.953－1.011) |
| No. of physicians* | 0.996 (0.979－1.015) | 0.993 (0.972－1.014) |
| Analysis using proportion of laborers |  |  |
| Proportion of laborers | 1.006 (0.980－1.032) | 0.998 (0.973－1.028) |
| Population density | 1.044 (1.009－1.079) | 1.021 (0.984－1.059) |
| Proportion of young people | 0.977 (0.937－1.021) | 1.002 (0.957－1.049) |
| No. of births* | 1.012 (0.975－1.051) | 0.997 (0.957－1.038) |
| Proportion of workers engaged in the secondary sector of industries | 1.003 (0.975－1.032) | 0.997 (0.966－1.028) |
| No. of designated emergency hospitals* | 1.023 (0.998－1.049) | 1.014 (0.985－1.042) |
| No. of medical clinics* | 0.992 (0.966－1.018) | 0.983 (0.955－1.012) |
| No. of physicians* | 0.997 (0.979－1.016) | 0.993 (0.973－1.014) |
| Analysis using proportion of unemployed persons |  |  |
| Proportion of unemployed persons | 1.038 (1.011－1.067) | 1.009 (0.982－1.039) |
| Population density | 1.038 (1.003－1.071) | 1.017 (0.978－1.050) |
| Proportion of young people | 0.979 (0.937－1.022) | 1.001 (0.959－1.056) |
| No. of births* | 1.015 (0.980－1.055) | 1.000 (0.958－1.040) |
| Proportion of workers engaged in the secondary sector of industries | 0.998 (0.970－1.027) | 0.995 (0.961－1.027) |
| No. of designated emergency hospitals* | 1.024 (0.999－1.048) | 1.015 (0.987－1.043) |
| No. of medical clinics* | 0.994 (0.971－1.021) | 0.983 (0.953－1.014) |
| No. of physicians* | 0.995 (0.976－1.013) | 0.993 (0.973－1.013) |
| Analysis using proportion of households living in owner–occupied housing |  |  |
| Proportion of households living in owner–occupied housing | 0.963 (0.935－0.994) | 0.974 (0.943－1.012) |
| Population density | 1.031 (0.991－1.069) | 1.008 (0.970－1.052) |
| Proportion of young people | 0.957 (0.908－1.001) | 0.988 (0.941－1.041) |
| No. of births* | 1.007 (0.970－1.047) | 0.994 (0.952－1.038) |
| Proportion of workers engaged in the secondary sector of industries | 1.012 (0.983－1.045) | 1.000 (0.968－1.037) |
| No. of designated emergency hospitals* | 1.019 (0.994－1.044) | 1.011 (0.983－1.039) |
| No. of medical clinics* | 0.985 (0.960－1.013) | 0.977 (0.949－1.006) |
| No. of physicians* | 0.994 (0.975－1.012) | 0.991 (0.970－1.012) |
| Analysis using proportion of divorced persons |  |  |
| Proportion of divorced persons | 1.066 (1.038－1.096) | 1.039 (1.009－1.071) |
| Population density | 1.032 (0.991－1.068) | 1.013 (0.981－1.059) |
| Proportion of young people | 0.982 (0.949－1.025) | 1.007 (0.951－1.050) |
| No. of births* | 1.010 (0.975－1.043) | 0.996 (0.960－1.045) |
| Proportion of workers engaged in the secondary sector of industries | 0.998 (0.970－1.025) | 0.992 (0.962－1.025) |
| No. of designated emergency hospitals* | 1.019 (0.994－1.042) | 1.011 (0.983－1.041) |
| No. of medical clinics* | 0.990 (0.965－1.017) | 0.981 (0.953－1.011) |
| No. of physicians* | 0.993 (0.975－1.012) | 0.992 (0.973－1.013) |
| CrI, credible intervals. Standardized values were used for all the explanatory variables. | | |
| * Number per 100,000 persons |  |  |
